# Efficacy of 1% carboxymethylcellulose sodium for treating dry eye after phacoemulsification: results from a multicenter, open-label, randomized, controlled study

**DOI:** 10.1186/s12886-015-0005-3

**Published:** 2015-03-20

**Authors:** Ke Yao, Yongzhen Bao, Jian Ye, Yi Lu, Hongsheng Bi, Xin Tang, Yune Zhao, Jinsong Zhang, Jinling Yang

**Affiliations:** Eye Center, Second Affiliated Hospital, Zhejiang University School of Medicine, Hangzhou, China; Peking University People’s Hospital, Beijing, China; Daping Hospital, Third Military Medical University, Chongqing, China; Eye, Ear, Nose and Throat Hospital of Fudan University, Shanghai, China; Shandong Shierming Eye Hospital, Jinan, China; Tianjin Eye Hospital, Tianjin, China; The Affiliated Eye Hospital of Wenzhou Medical College, Wenzhou, China; The Fourth Affiliated Hospital of China Medical University, Shenyang, China; Allergan Information Consulting (Shanghai) Co, Ltd, Shanghai, China

**Keywords:** Carboxymethylcellulose, Dry eye, Phacoemulsification, Cataract surgery

## Abstract

**Background:**

For patients who experience dry eye after phacoemulsification, vision and quality of life can significantly deteriorate. In this study, the efficacy and safety of carboxymethylcellulose sodium (CMC) 1% ophthalmic solution combined with conventional therapy in treating dry eye signs and symptoms after phacoemulsification were evaluated.

**Methods:**

In this prospective, multicenter, open-label, controlled study, 180 patients with age-related cataract were randomized to treatment with conventional therapy plus CMC 1% (n = 90) or to conventional therapy only (control group, n = 90) after phacoemulsification and intraocular lens implantation. Tear breakup time (TBUT), the Schirmer test with anesthesia, and fluorescein and lissamine green staining were performed. The Ocular Surface Disease Index (OSDI) questionnaire and a patient subjective symptom evaluation were administered preoperatively (baseline) and postoperatively at 7 and 30 days.

**Results:**

TBUT was significantly longer in the treatment group compared with the control group at day 7 (8.5 ± 5.5 versus 6.6 ± 3.8 s; *P* = 0.0475) and day 30 (9.0 ± 5.9 versus 6.7 ± 4.8 s; *P* = 0.0258) after surgery. Compared with baseline, TBUT significantly increased in patients in the treatment group (*P* < 0.001 at both day 7 and day 30) with a presurgical diagnosis of dry eye, but significantly decreased in patients in the control group (*P* < 0.02 at both day 7 and day 30) with no prior diagnosis of dry eye. Fluorescein and lissamine staining, OSDI questionnaire and subjective symptom scores all improved from baseline, with no significant differences between the two groups. No significant differences in tolerability and safety were observed between the group receiving CMC and conventional therapy, and those receiving conventional therapy only.

**Conclusion:**

Treatment with CMC 1% can provide significant improvement in tear film stability after phacoemulsification for age-related cataract.

**Trial registration:**

ClinicalTrials.gov identifier NCT02028754 (Date of registration: Jan. 6, 2014).

## Background

Dry eye is a multifactorial disease of the tears and ocular surface that results in symptoms of discomfort, visual disturbance, and tear film instability, with potential damage to the ocular surface [1,2]. Highly prevalent worldwide, dry eye has several risk factors, including age, contact lens wear, diet, smoking, history of allergy or diabetes, and activities that involve prolonged and demanding visual tasks (eg, use of computers, smart phones, and other devices) [3]. Quality of life may be significantly impacted with the conduct of daily activities such as reading, driving, and work using computer screens potentially affected [4].

With the advancement of medical technology, cataract surgery (phacoemulsification with intraocular lens [IOL] implantation) has become a routine procedure; however, for patients who experience dry eye after phacoemulsification, vision and quality of life can significantly deteriorate. Clinical evaluation of dry eye after cataract surgery has demonstrated worsening of patient subjective symptom scores, reductions in tear film breakup time (TBUT) and goblet cell density, as well as increased corneal and conjunctival staining, indicating deteriorating disease [5-7]. Various factors may further impact the health of the ocular surface following cataract surgery, including age, the external environment, poor systemic health, concurrent ocular surface disease, reflex secretory block caused by nerve injury, and ocular epithelial injury during the operation [1,8]. Increased ocular discomfort reported after cataract surgery may be a result of failure to diagnose dry eye before surgery and/or subsequent inadequate treatment after cataract surgery.

Conventional therapy following cataract surgery includes topical corticosteroids or non-steroidal anti-inflammatory drugs to manage inflammation, and anti-infectives. The effectiveness of corticosteroids in managing dry eye after cataract surgery has been evaluated [9,10]. However, few studies have assessed the addition of artificial tears to conventional therapy for treatment of dry eye after cataract surgery [11]. The objective of this study was to investigate the efficacy and safety of carboxymethylcellulose sodium (CMC) 1% ophthalmic solution added to conventional therapy in stabilizing tear film and treating preexisting dry eye or dry eye resulting from phacoemulsification.

## Methods

This prospective, open-label, interventional, randomized, controlled study was conducted at eight clinical sites in China between October 2011 and May 2013. The study protocol was approved by the central ethics committee (Ethics Committee of the Second Hospital of Zhejiang Medical University) and two branch ethics committees (Ethics Committee of Daping Hospital of The Third Military Medical University and Ethics Committee of Eye, Ear, Nose and Throat Hospital of Fudan University). The study was conducted in accordance with the principles of the Declaration of Helsinki, and all patients signed an informed consent form before enrollment.

### Patients

Male or female patients aged 60 to 80 years with age-related cataract were enrolled. Patients were scheduled to undergo phacoemulsification and IOL implantation and had a lens nucleus hardness of grade 3 or less (based on nucleus color per the standard Lens Opacities Classification System III [12]). Excluded were patients with allergy to any of the study medications, conjunctival allergy or infectious disease, history of ocular chemical or thermal burn, Stevens-Johnson syndrome or ocular pemphigoid, glaucoma or ocular hypertension, eyelid or lacrimal disease, any ocular operation within 3 months prior to enrollment, corneal contact lens wear, history of serious systemic disease, or other conditions that in the opinion of the investigator precluded enrollment. Patients were withdrawn from the study if they experienced complications during surgery, or post-surgical ocular hypertension, endophthalmitis, infectious keratitis, or conjunctivitis.

### Randomization and treatment administration

Patients who provided informed consent were enrolled in the study by their treating physician, and were assigned a sequential number with a corresponding randomization code generated by an independent third party using SAS software (version 8.0, SAS Institute Inc, Cary, NC). According to the randomization code, clinical staff assigned patients to receive either study treatment plus conventional post-surgical therapy consisting of (1) prednisolone acetate ophthalmic suspension 1% (Pred Forte®, Allergan, Inc., Irvine, CA) instilled four times daily in the first post-surgical week, three times daily in the second week, twice daily in the third week, and once daily for the remainder of the study, and (2) levofloxacin 0.5% ophthalmic solution (Cravit®, Santen Pharmaceutical Co, Ltd, Osaka, Japan), or conventional therapy alone (control group) instilled four times daily in the first post-surgical week. The clinical staff provided treatment medications and instructions on how to administer ophthalmic solutions according to the assigned randomization group.

Topical anesthesia with oxybuprocaine hydrochloride 0.4% eye drops (Benoxil, Santen Pharmaceutical Co, Ltd, Osaka, Japan) or proparacaine hydrochloride 0.5% eye drops (Alcaine®, Alcon Laboratories, Inc, Ft Worth, TX) was administered before the surgical procedure. After surgery, the study treatment group instilled CMC sodium 1% ophthalmic solution (Refresh Liquigel®, Allergan, Inc., Irvine, CA) four times daily, along with conventional post-surgical therapy for the first 30 days. The control group received conventional post-surgical therapy only. During the study, use of other topical ophthalmologic agents with a potential impact on efficacy assessment of the study treatment was prohibited.

### Clinical assessments

Patients were evaluated 3 to 5 days before phacoemulsification and IOL implant surgery (baseline), as well as 7 and 30 days post-surgery to assess the benefit of CMC 1% at early and later time points. On the day of each of the three visits, administration of CMC was withheld prior to and immediately after study assessments. The following were conducted in sequence: TBUT, fluorescein and lissamine green ocular staining, and Schirmer test with oxybuprocaine hydrochloride 0.4% eye drops (Benoxil, Santen Pharmaceutical Co, Ltd) or proparacaine hydrochloride 0.5% eye drops (Alcaine^®^, Alcon Laboratories, Inc) as anesthetics (and test strips from Tianjin Jingming New Technological Development Co, Ltd, Tianjin, China). The Ocular Surface Disease Index (OSDI) questionnaire and patient subjective symptom evaluation were also completed at each visit, prior to the above tests.

The primary efficacy outcome, TBUT, was measured in seconds after placing a fluorescein sodium test strip on the lower eye lid. Fluorescein staining was conducted to assess corneal epithelial damage and was graded on a 4-point scale in three corneal regions: upper, middle, and lower. Corneal and conjunctival staining were also performed by placing a lissamine green test strip (Intra Ocular Care Pvt. Ltd, Bangalore, India) on the lower eye lid. Five regions, the temporal and nasal conjunctival regions and three corneal regions (upper, middle, and lower), were assessed. Corneal and conjunctival were graded in each region as 0 = no staining; 1 = staining less than half the area; 2 = staining an area greater than half but not the whole region; and 3 = staining of the whole region. The Schirmer test was performed to evaluate basic secretion from the lacrimal glands [13].

The standard OSDI questionnaire and a patient subjective symptom evaluation form were used to evaluate dry eye symptoms. In the OSDI questionnaire, 12 questions assessed visual function, ocular symptoms, and potential environmental triggers. The subjective evaluation form included 11 symptoms: foreign body sensation, photophobia, itching, pain in the eyes, dry eyes, eye heaviness, blurred vision, eye fatigue, eye discomfort, eye secretions, and tearing. Each symptom was graded as 0 = no symptom; 1 = occasional symptoms; 2 = intermittent mild symptoms; and 3 = persistent clear symptoms.

The clinical investigator was responsible for monitoring adverse events (eg, eye swelling, ocular hypertension) and reporting incidents to the study sponsor. Any worsening of pre-existing dry eye symptoms (eg, tearing, foreign body sensation, or photophobia) or new occurrence of such symptoms was also considered an adverse event.

### Data analysis and statistical methods

Patients were diagnosed as having dry eye symptoms if they met one of the following conditions: TBUT ≤5 s, plus a subjective symptom score of 3 or greater [14,15], or Schirmer test ≤3 mm in 5 min, plus a subjective symptom score of 3 or greater [14,16]. The effectiveness of treatment with 1% CMC in relieving dry eye symptoms was assessed by comparing differences in clinical assessments between the treatment group and control group. Pre- and post-surgery differences were also determined within the treatment and control groups. Subgroup analyses were performed in patients with and without a diagnosis of dry eye before surgery.

Quantitative data were compared between the two groups using the *t*-test (normal distribution) and the Wilcoxon rank sum test (non-normal distribution). Similarly, differences before and after surgery within the treatment and control groups were assessed using paired *t*-tests (normal distribution) and Wilcoxon signed rank test (non-normal distribution). *P* < 0.05 was considered to be statistically significant. Statistical analysis was performed using SAS (version 8.0).

Sample size for the study was based on results from an early study, in which the value of TBUT was determined to be 4.2 ± 2.7 s in the control group and 7.3 ± 4.9 s in the artificial tear group at day 7 after cataract surgery [17]. One-tailed test was conducted with α = 0.05 and β = 0.2, and it was determined that at least 31 patients per group were required to detect superiority between the treatment groups. Taking patient discontinuation into consideration, as well as post-surgery follow-up visits at both day 7 and day 30, it was estimated that a sample size of 90 patients in each group was required for the intent-to-treat (ITT) population.

## Results

**Figure 1:**
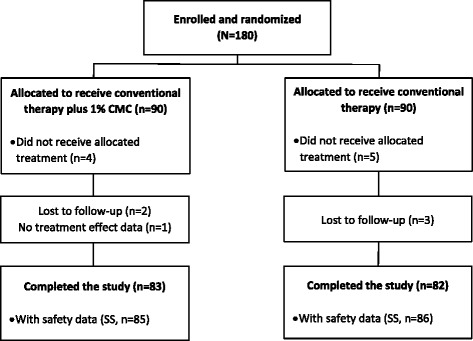
**Patient disposition.** ITT, intent to treat; CMC, carboxymethylcellulose sodium; SS, safety set.

A total of 180 patients were enrolled and formed the ITT population—90 patients were randomized to each study group. Among the enrolled patients, seven patients in the treatment group and eight patients in the control group did not complete the study (Figure 1). Baseline characteristics were well balanced between the two groups (Table 1). Overall, 52.3% and 47.7% of patients in the treatment group had surgery in the right and left eye, compared with 55.5% and 44.4% in the control group, respectively. The percentage of patients who had a superior (12 o’clock), temporal (9 o’clock), tempo-superior (10–11 or 11–12 o’clock), or nasal superior (10–11 or 11–12 o’clock) incision was also similar between both groups. The mean incision length (± SD) was 2.8 ± 0.3 cm versus 2.8 ± 0.4 cm in the treatment and control groups, respectively.

**Table 1:** **Baseline demographics and disease characteristics**

**Baseline characteristic**	**Treatment group (n = 90)**	**Control group (n = 90)**	***P*** **value**
Age, years (mean ± SD)	68.8 ± 7.1	69.0 ± 7.1	0.9349*
Female, n (%)	54 (60.0)	59 (65.6)	0.4407**
Patient with concomitant systemic diseases, n (%)	25 (27.8)	26 (28.9)	0.8686**
TBUT, s (mean ± SD)	7.7 ± 4.8	7.2 ± 4.1	0.7583*
Schirmer test, mm (mean ± SD)	12.6 ± 6.9	12.9 ± 6.9	0.6800*
Percentage of patients with dry eye, n (%)			
TBUT ≤5 s + symptom score ≥3	34 (37.8)	32 (35.6)	
Schirmer test ≤3 mm + symptom score ≥3	2 (2.2)	4 (4.4)	
Total patients with dry eye	35 (38.9)	33 (36.7)	0.7585**

SD, standard deviation; TBUT, tear film breakup time.

*Wilcoxon rank sum test.

**Chi-square test.

### Dry eye diagnosis before and after surgery

**Figure 2:**
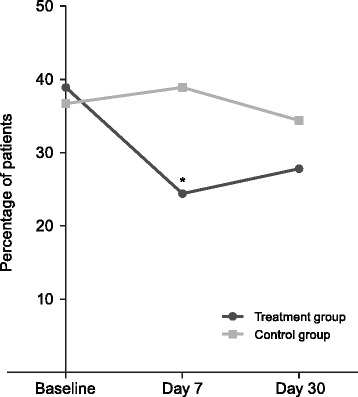
**Percentage of patients with dry eye according to study criteria, at baseline and at days 7 and 30 after cataract surgery.** *P = 0.0373 compared with control group.

Based on the criteria for diagnosis of dry eye in this study, the percentage of patients considered to have dry eye disease before surgery was similar in both the treatment and control groups. At day 7 after surgery, the number of patients with dry eye decreased to 22 (24.4%) in the treatment group but increased slightly to 35 (38.9%) in the control group (*P* = 0.0373 between groups) (Figure 2). By day 30 following cataract surgery, 25 (27.8%) patients in the treatment group and 31 (34.4%) in the control group had dry eye (*P* = 0.3340 between groups).

Among the group of patients with dry eye before surgery, a significantly lower number of patients continued to have dry eye after surgery in the treatment group (16; 45.7%) compared with the control group (25; 75.8%) at day 7 (*P* = 0.0114). At day 30, no significant differences were found in the number of patients with post-surgical dry eye in the two groups (17 [48.6%] versus 20 [60.6%]). In the subgroup of patients without dry eye before surgery, no differences were observed between the two groups at both day 7 (6 [10.9%] versus 10 [17.5%]) and day 30 (8 [14.5%] versus 11 [19.3%]) post-surgery.

### Tear breakup time

**Figure 3:**
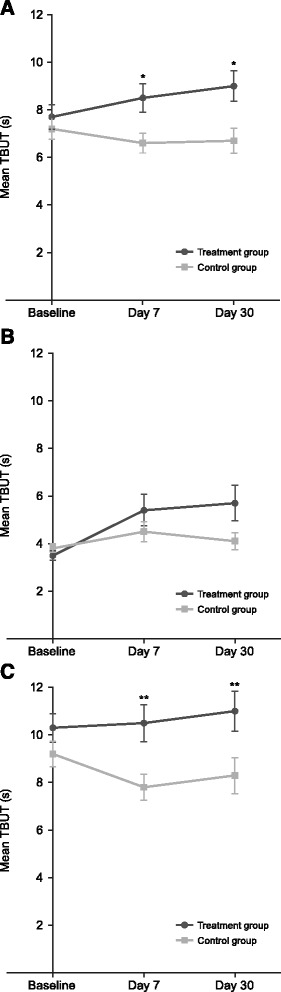
**Tear film breakup time (TBUT) at baseline and at days 7 and 30 after surgery. (A)** All patients undergoing cataract surgery. **(B)** Subgroup of patients with dry eye before cataract surgery. **(C)** Subgroup of patients without dry eye before cataract surgery. *P ≤ 0.0475; **P ≤ 0.0168 compared with control group; error bars represent standard error mean.

After cataract surgery, TBUT (mean ± SD) was significantly greater in the treatment group than the control group at both day 7 and day 30 (Figure 3A; Table 2). Compared with baseline, TBUT increased at days 7 and 30 in the treatment group, but decreased at both time points in the control group (Table 2).

**Table 2:** **TBUT at baseline and days 7 and 30 after cataract surgery**

**TBUT in patient subgroups**	**Treatment group**		**Control group**		***P*** value*
	**n**	**Score**	**n**	**Score**	
All patients, seconds (mean ± SD)					
Baseline	90	7.7 ± 4.8	90	7.2 ± 4.1	0.7583
At day 7	85	8.5 ± 5.5	85	6.6 ± 3.8	**0.0475**
At day 30	83	9.0 ± 5.9	82	6.7 ± 4.8	**0.0258**
Change from baseline at day 7	85	0.9 ± 4.9	85	−0.5 ± 3.4**	**0.0171**
Change from baseline at day 30	83	1.3 ± 5.6**	82	−0.4 ± 4.4**	**0.0093**
Dry eye prior to surgery subgroup, s (mean ± SD)					
Baseline	35	3.5 ± 1.2	33	3.8 ± 1.0	0.2912
At day 7	33	5.4 ± 3.8	32	4.5 ± 2.4	0.5064
At day 30	32	5.7 ± 4.2	31	4.1 ± 2.0	0.3146
Change from baseline at day 7	33	2.0 ± 3.6***	32	0.7 ± 2.2	0.0805
Change from baseline at day 30	32	2.3 ± 3.8***	31	0.3 ± 2.0	**0.0365**
Non-dry eye prior to surgery subgroup, s (mean ± SD)					
Baseline	55	10.3 ± 4.4	57	9.2 ± 4.0	0.1283
At day 7	52	10.5 ± 5.6	53	7.8 ± 4.0	**0.0168**
At day 30	51	11.0 ± 6.0	51	8.3 ± 5.4	**0.0164**
Change from baseline at day 7	52	0.1 ± 5.5	53	−1.3 ± 3.8**	0.1300
Change from baseline at day 30	51	0.7 ± 6.4	51	−0.9 ± 5.3**	0.1501

TBUT, tear breakup time; SD, standard deviation.

*P values for the difference between groups calculated using Wilcoxon rank sum test.

**P ≤ 0.0349; ***P ≤ 0.0007 for change from baseline calculated using Wilcoxon signed rank test.

Significant differences between treatment groups and significant changes from baseline are indicated in bold font.

Subgroup analysis in patients diagnosed with dry eye prior to cataract surgery demonstrated no statistically significant differences in TBUT between the treatment group and control group at days 7 and 30 (Figure 3B; Table 2). However, there was a statistically significant increase from baseline in TBUT in the treatment group (*P* < 0.001). In contrast, no improvements in TBUT were observed in the control group up to 30 days after surgery (Table 2). Among patients without dry eye prior to cataract surgery, significant differences were observed in TBUT between the treatment and control groups at days 7 and 30 (Figure 3C; Table 2). In the control group, there was a statistically significant decrease from baseline in TBUT at both time points. In the treatment group, no significant changes were noted from baseline at both day 7 and day 30 (Table 2).

### Schirmer test

No significant differences were observed in the Schirmer test (mean ± SD) between the two groups at both day 7 and day 30 after cataract surgery (Table 3). No significant changes in Schirmer test scores were reported in either group at day 7 or day 30 compared with baseline. No differences were observed in subgroup analyses of patients with dry eye or those without dry eye prior to cataract surgery.

**Table 3:** **Schirmer test, fluorescein staining, and lissamine green staining at baseline and after cataract surgery**

**Clinical assessment**	**Treatment group**		**Control group**		***P*** **value** *
	**n**	**Score**	**n**	**Score**	
Schirmer test, mm (mean ± SD)					
Baseline	90	12.6 ± 6.9	90	12.9 ± 6.9	0.6800
At day 7	85	11.8 ± 5.6	85	12.5 ± 6.8	0.6620
At day 30	83	12.7 ± 6.1	82	12.6 ± 6.5	0.6929
Change from baseline at day 7	85	−0.8 ± 5.9	85	−0.3 ± 5.1	0.9055
Change from baseline at day 30	83	0.0 ± 5.4	82	−0.1 ± 5.6	0.6889
Fluorescein staining score (mean ± SD)					
Baseline	90	2.0 ± 2.8	90	1.7 ± 2.6	0.4084
At day 7	85	2.0 ± 2.8	85	1.8 ± 2.6	0.7092
At day 30	83	1.1 ± 1.8	82	1.2 ± 1.9	0.3984
Change from baseline at day 7	85	0.0 ± 1.7	85	0.0 ± 1.9	0.5236
Change from baseline at day 30	83	−1.0 ± 2.3****	82	−0.6 ± 2.0***	0.2207
Lissamine staining score (mean ± SD)					
Baseline	90	2.9 ± 3.2	90	2.7 ± 3.1	0.6162
At day 7	85	2.9 ± 3.3	85	2.6 ± 3.2	0.7955
At day 30	83	2.1 ± 2.4	82	2.3 ± 2.6	0.4380
Change from baseline at day 7	85	−0.2 ± 2.5	85	−0.3 ± 2.8	0.7685
Change from baseline at day 30	83	−1.0 ± 2.6****	82	−0.6 ± 2.6**	0.2404

SD, standard deviation.

*P values for the difference between groups calculated using Wilcoxon rank sum test.

**P = 0.0268; ***P = 0.0070; ****P ≤ 0.0004 for change from baseline calculated using Wilcoxon signed rank test.

Significant changes from baseline are indicated in bold font.

### Fluorescein and lissamine green staining

No differences in fluorescein staining scores (mean ± SD) were observed between the treatment and control groups at either day 7 or day 30 post-surgery. However, fluorescein scores significantly decreased from baseline at day 30 in the treatment and control groups (Table 3). No significant improvements compared with baseline were observed among patients with dry eye before cataract surgery. For patients without dry eye before surgery, fluorescein staining score at day 30 post-surgery decreased significantly from baseline in the treatment group (2.1 ± 3.1 to 1.0 ± 1.8; *P* < 0.001); no significant changes were observed in the control group.

Lissamine green staining scores (mean ± SD) were not significantly different between the two groups at day 7 and day 30 post-surgery. Similar to fluorescein scores, there were significant decreases from baseline in lissamine green scores at day 30 in both the treatment and control groups (Table 3). Among patients with dry eye before surgery, no significant improvements were observed in lissamine green staining at day 7 and day 30 post-surgery. For patients without dry eye prior to surgery, lissamine green scores significantly decreased from baseline at day 7 in the control group (2.8 ± 2.9 to 2.2 ± 2.4; *P* = 0.0233) and at day 30 in the treatment group (3.1 ± 3.5 to 2.1 ± 2.4; *P* = 0.0009).

### OSDI questionnaire and patient subjective symptom evaluation

No differences were reported in OSDI and patient subjective symptom scores (mean ± SD) between the treatment group and control group at day 7 and day 30 following cataract surgery. However, both OSDI and subjective symptom scores significantly decreased from baseline at day 7 and day 30 post-cataract surgery in both groups (Table 4). In subgroup analyses of patients with and without dry eye before cataract surgery, no significant differences in mean OSDI and subjective symptom scores were observed between the treatment and control groups at day 7 and day 30 post-surgery. Compared with baseline, there were significant decreases in OSDI and symptom scores within the treatment group and control group at both days 7 and 30 post-surgery. Reductions in the scores were not significantly different between the groups, except for the mean subjective symptom score at day 30 after cataract surgery in patients without dry eye (12.1 ± 6.5 to 4.8 ± 3.5 in the treatment group versus 11.0 ± 5.7 to 5.5 ± 4.0 in the control group; *P* = 0.0466).

**Table 4:** **OSDI questionnaire and patient subjective symptom evaluation scores at baseline and after cataract surgery**

**Subject assessment**	**Treatment group**		**Control group**		***P*** value*
	**n**	**Score**	**n**	**Score**	
OSDI questionnaire score (mean ± SD)					
Baseline	90	41.8 ± 20.8	90	44.8 ± 20.9	0.2116
At day 7	85	15.6 ± 16.8	85	18.1 ± 19.5	0.4934
At day 30	83	12.7 ± 11.3	82	14.2 ± 13.8	0.7243
Change from baseline at day 7	85	−26.7 ± 20.3**	85	−27.2 ± 22.7***	0.7447
Change from baseline at day 30	83	−29.9 ± 19.4***	82	−30.8 ± 20.6***	0.7748
Subjective symptom evaluation score (mean ± SD)					
Baseline	90	11.8 ± 6.2	90	11.5 ± 6.0	0.7700
At day 7	85	6.1 ± 5.3	85	6.4 ± 5.2	0.4737
At day 30	83	5.1 ± 3.6	82	5.6 ± 4.0	0.5971
Change from baseline at day 7	85	−5.9 ± 5.2**	85	−5.4 ± 5.6***	0.9018
Change from baseline at day 30	83	−7.0 ± 4.7**	82	−6.2 ± 5.8***	0.6069

OSDI, Ocular Surface Disease Index; SD, standard deviation.

*P values for the difference between groups calculated using Wilcoxon rank sum test except for OSDI. Change from baseline at day 30 calculated using *t*-test.

**P < 0.0001 for change from baseline calculated using Wilcoxon signed rank test.

***P < 0.0001 for change from baseline calculated using paired *t*-test.

Significant changes from baseline are indicated in bold font.

### Safety

A total of 171 patients, 86 in the treatment group and 85 in the control group, formed the safety analysis set. No significant differences were noted in safety between the groups; 5 (5.8%) patients in the treatment group and 4 (4.7%) patients in the control group experienced adverse events (Table 5). The most common adverse events reported were high intraocular pressure in the treatment group, and foreign body sensation in the control group (Table 5).

**Table 5:** **Adverse events reported by patients in either the treatment group or control group**

**Adverse event, n (%)**	**Treatment group (n = 86)**	**Control group (n = 85)**
High intraocular pressure	3 (3.5)	1 (1.2)
Tearing	1 (1.2)	1 (1.2)
Eye swelling	1 (1.2)	1 (1.2)
Foreign body sensation	1 (1.2)	2 (2.4)
Photophobia	0 (0)	1 (1.2)
Red eye	0 (0)	1 (1.2)
Impaired vision	0 (0)	1 (1.2)

## Discussion

Dry eye is a common condition following cataract surgery and can be a significant cause of vision impairment, reducing patients’ quality of life. In this study, 37.78% of all patients were diagnosed with dry eye at baseline based on the study criteria. The proportion was higher than the 20% to 30% that has been reported for the general population in China [18,19].

Aging is an established risk factor for dry eye [3], and androgen deficiency associated with aging has also been reported to be a contributing factor to dry eye [20,21]. The patient population enrolled in this study, aged 60 to 80 years with age-related cataract, was at greater risk of dry eye disease. Patients with TBUT ≤5 s, plus a subjective symptom score of ≥3, constituted 36.67% of the total patient population, and 97.06% of patients who enrolled in the study had been diagnosed with dry eye before phacoemulsification. This observation indicates that a majority of patients with dry eye prior to cataract surgery may have poor tear film stability and accompanying symptoms.

This study demonstrated that by day 7 after phacoemulsification, the percentage of patients with dry eye decreased from 38.9% to 24.4% in the treatment group, but remained relatively stable (36.7%-38.9%) in the control group. The findings indicate that cataract surgery may worsen dry eye (as has been previously reported), and further suggest that the addition of CMC 1% to conventional post-surgical therapy may help prevent development/worsening of dry eye after phacoemulsification. Since neither patients nor investigators were masked to treatment, patients in the control group were likely aware of their treatment status, which could explain the above findings. However, despite this potential limitation of the study, the difference in the percentage of patients with dry eye between the treatment and control groups was not significant by day 30 after surgery, arguing against this possibility. One possible, alternative explanation for this observation is that the ocular surface may have recovered from damage caused by phacoemulsification. Consistent with this possibility, the post-surgical decrease in symptoms observed in the treatment and control groups may be attributable to enhanced vision as a result of phacoemulsification, although it is also possible that the questionnaires used were not optimally designed to assess dry eye following cataract surgery.

CMC is a polymer composed of glucopyranose subunits with anionic charge and high microviscosity properties, which allow retention on the cornea for a prolonged time period. An in vitro study reported that CMC may remain bound to human corneal epithelial cells (HCECs) for 2 h, and that CMC may bind to HCECs through interactions between glucopyranose subunits and glucose transporters [22]. A single-center, double-masked, randomized, crossover study also demonstrated that CMC 1% ophthalmic solution prolonged TBUT and improved the Ocular Protection Index for at least 20 min after instillation [23]. Since reductions in TBUT after cataract surgery have been reported previously [6,7], administering artificial tears to stabilize the tear film may thus help prevent the development/worsening of dry eye after phacoemulsification.

It is unclear why post-surgical TBUT values observed in this study were similar in the treatment and control groups of patients who had dry eye prior to surgery. However, since steroids can reduce inflammation in the injured ocular surface epithelium of patients with dry eye, and prednisolone was part of the post-surgical therapy administered to the entire study population, it is possible that prednisolone prevented the detection of a difference between the treatment and control groups. Nonetheless, at day 30 post-cataract surgery, TBUT decreased from baseline in the control group, but improved in the treatment group. This finding is consistent with that of a previous study, which demonstrated that CMC prolonged TBUT in patients with mild to moderate dry eye [24]. Our study also showed that in patients with a previous diagnosis of dry eye, CMC 1% prolonged TBUT at days 7 and 30 post-surgery (compared with the control group). Among patients without dry eye prior to surgery, CMC 1% maintained normal TBUT at days 7 and 30 post-surgery; in contrast, a decrease in TBUT was observed in the control group. These results thus support a stabilizing effect of CMC 1% ophthalmic solution on the tear film.

As increasing evidence supports the hypothesis that ocular surface and lacrimal gland inflammation plays a central role in the pathogenesis of dry eye [25,26], the use of postsurgical prednisolone therapy in our study could also explain why corneal and conjunctival staining decreased in both the treatment and control groups at 30 days post-surgery. For patients without dry eye prior to cataract surgery, fluorescein staining and lissamine staining scores in the treatment group decreased significantly compared with baseline. In contrast, improvement in epithelial staining was not observed in the control group. These results suggest that the addition of CMC 1% may help epithelial recovery following cataract surgery, consistent with other studies in which CMC was shown to promote corneal epithelial wound closure in both in vitro and animal models [22,27].

## Conclusions

Although this study was limited in that neither patients nor investigators were masked to treatment, results indicate that adding CMC 1% ophthalmic solution to conventional therapy after phacoemulsification can help manage patients with prior dry eye disease. This combination can also maintain tear film stability and TBUT in patients without dry eye disease prior to phacoemulsification. Further studies are required, designed to determine mechanisms in which CMC helps stabilize the tear film after cataract surgery.

## Abbreviations

CMC: Carboxymethylcellulose
HCEC: Human corneal epithelial cells
IOL: Intraocular lens
OSDI: Ocular Surface Disease Index
SD: Standard deviation
TBUT: Tear breakup time

## References

[1] International Dry Eye Workshop (2007). The definition and classification of dry eye disease: report of the Definition and Classification Subcommittee of the International Dry Eye WorkShop (2007). Ocul Surf.

[2] Miljanovic B, Dana R, Sullivan DA, Schaumberg DA (2007). Impact of dry eye syndrome on vision-related quality of life. Am J Ophthalmol.

[3] International Dry Eye Workshop (2007). The epidemiology of dry eye disease: report of the Epidemiology Subcommittee of the International Dry Eye WorkShop (2007). Ocul Surf.

[4] Friedman NJ (2010). Impact of dry eye disease and treatment on quality of life. Curr Opin Ophthalmol.

[5] Han KE, Yoon SC, Ahn JM, Nam SM, Stulting RD, Kim EK (2014). Evaluation of dry eye and meibomian gland dysfunction after cataract surgery. Am J Ophthalmol.

[6] Li XM, Hu L, Hu J, Wang W (2007). Investigation of dry eye disease and analysis of the pathogenic factors in patients after cataract surgery. Cornea.

[7] Oh T, Jung Y, Chang D, Kim J, Kim H (2012). Changes in the tear film and ocular surface after cataract surgery. Jpn J Ophthalmol.

[8] Movahedan A, Djalilian AR (2012). Cataract surgery in the face of ocular surface disease. Curr Opin Ophthalmol.

[9] Chung YW, Oh TH, Chung SK (2013). The effect of topical cyclosporine 0.05% on dry eye after cataract surgery. Korean J Ophthalmol.

[10] Roberts CW, Elie ER (2007). Dry eye symptoms following cataract surgery. Insight.

[11] Sanchez MA, Arriola-Villalobos P, Torralbo-Jimenez P, Giron N, de la Heras B, Herrero Vanrell R (2010). The effect of preservative-free HP-Guar on dry eye after phacoemulsification: a flow cytometric study. Eye (Lond).

[12] Chylack LT, Wolfe JK, Singer DM, Leske MC, Bullimore MA, Bailey IL (1993). The Lens Opacities Classification System III. The Longitudinal Study of Cataract Study Group. Arch Ophthalmol.

[13] Foulks GN (2003). Challenges and pitfalls in clinical trials of treatments for dry eye. Ocul Surf.

[14] Wang Z, Wang H, Xu W, Bi Y-L (2007). The changes of tear film after phacoemulsification combining with intraocular lens implantation. J Tongji Univer (Medi Sci).

[15] Li H, Yuan F (2005). The effects of phacoemulsification on tear film and eye surface. Chin J Optom Ophthalmol Vis Sci.

[16] International Dry Eye Workshop (2007). Methodologies to diagnose and monitor dry eye disease: report of the Diagnostic Methodology Subcommittee of the International Dry Eye WorkShop (2007). Ocul Surf.

[17] Lu B, Zhang JS (2007). The effect of artificial tears on tear film changes after phacoemulsification and intraocular lens implantation. Int J Ophthalmol.

[18] Zhang H, An X, Chen X-Y (2008). Prevalence and risk factors associated with dry eye in a hospital-based population. Rec Adv Ophthalmol.

[19] Tian Y-J, Liu Y, Zou H-D, Jiang Y-J, Liang X-Q, Sheng M-J (2009). Epidemiologic study of dry eye in population of over 20 years old in Jiangning District of Shanghai. Chinese J Ophthalmol.

[20] Cermak JM, Krenzer KL, Sullivan RM, Dana MR, Sullivan DA (2003). Is complete androgen insensitivity syndrome associated with alterations in the meibomian gland and ocular surface?. Cornea.

[21] Sullivan BD, Evans JE, Dana MR, Sullivan DA (2006). Influence of aging on the polar and neutral lipid profiles in human meibomian gland secretions. Arch Ophthalmol.

[22] Garrett Q, Simmons PA, Xu S, Vehige J, Zhao Z, Ehrmann K (2007). Carboxymethylcellulose binds to human corneal epithelial cells and is a modulator of corneal epithelial wound healing. Invest Ophthalmol Vis Sci.

[23] Simmons PA, Vehige JG (2007). Clinical performance of a mid-viscosity artificial tear for dry eye treatment. Cornea.

[24] Lee JH, Ahn HS, Kim EK, Kim TI (2011). Efficacy of sodium hyaluronate and carboxymethylcellulose in treating mild to moderate dry eye disease. Cornea.

[25] Pflugfelder SC, Corrales RM, de Paiva CS (2013). T helper cytokines in dry eye disease. Exp Eye Res.

[26] Stevenson W, Chauhan SK, Dana R (2012). Dry eye disease: an immune-mediated ocular surface disorder. Arch Ophthalmol.

[27] Garrett Q, Xu S, Simmons PA, Vehige J, Xie RZ, Kumar A (2008). Carboxymethyl cellulose stimulates rabbit corneal epithelial wound healing. Curr Eye Res.

